# On Growing Old

**Published:** 1958-07

**Authors:** J. H. Sheldon

**Affiliations:** Senior Physician, the Royal Hospital, Wolverhampton


### ON GROWING OLD

BY

J. H. SHELDON, C.B.E., M.D., F.R.C.P.

*Senior Physician, The Royal Hospital, Wolverhampton*

THE FORTY-SIXTH LONG FOX MEMORIAL LECTURE DELIVERED ON 22ND OCTOBER, 1957

My first duty is to express my sincere appreciation of the great honour of your invitation to deliver the Edward Long Fox Memorial Lecture, which has given me all the greater pleasure in that it enables me to renew an acquaintance with a City—and a University—with which I have had such pleasant associations. I want to use the opportunity to discuss with you some of the problems of ageing, both subjective and objective, individual and social, but I must hasten to make it clear that my approach will be descriptive rather than hortatory, and that it is no part of my purpose to offer advice, except at times by implication, on how to grow old. I should indeed feel venturesome in doing so, for I am not yet officially old myself, though I regret to say that duration for a further 338 days will entitle me to claim full membership of that category. This leads me to make a point which is often forgotten—that so much advice is given concerning the problems of the old and so many decisions are taken on their behalf, by individuals who have yet to grow old themselves, and therefore do not know it from the inside. This has, in fact, been voiced with great effect by one of the most remarkable old people in these islands—Miss Margery Fry[1]. She makes the point that one result of the rarity of really old people in the past is that in literature, while younger people are treated as individuals, the older tend to be treated more impersonally as types—and she instances such a common stage direction as "enter an old man". This is a valid point, with clinical and scientific as well as literary implications, and it is to be hoped that the increase in the supply of old people may allow them to contribute effectually to the literature of their own subject.

### INCREASING NUMBERS OF OLD PEOPLE

I shall begin by discussing ageing from the general point of view, as a population change. The geographical aspects of this change in population structure are among its most interesting features. In a human community with a high fertility rate and a high mortality rate, the proportion of individuals of 65 years and over in the population would seem to be usually around 2 per cent. of the whole, at which level the social stresses imposed by age can be carried without undue difficulty. This figure tends to be remarkably stable and improvements in mortality rates only affect it very slowly. Thus in Ceylon the proportion of old people in 1881 was 2 per cent. and it had only risen to 3·2 per cent. by 1951. By contrast, there were in 1954, six countries whose population contained 10 per cent. or more of old people, and all of these were in Western Europe. France headed the list with some 12 per cent.—or nearly one person in eight—and the others were Belgium, Great Britain, Eire, Sweden and Eastern Germany[2]. At this level the stresses imposed on the community by its old people raise problems sufficient to become matters of public concern. Following these come a group of countries with a ratio of old people lying between 1 in 15 ($6\frac{2}{3}$ per cent.) and 1 in 10. They have an enormous total population, for the group includes the whole of Western Europe outside the Iron Curtain, the great dominions of Canada, Australia and New Zealand, and the United States of America. So far as is known, most if not all other countries have an old-age population ratio of less than 1 in 15. There are several things to be said about the bare facts listed above. In the first place, the position is not static and we are in fact witnessing a population change in active progress at the present moment, steadily increasing the proportion of old people. Thus in Norway a proportion of 9 per cent. in 1954 is expected to rise to at least 15 per cent. by 1975. In the next place it is important to remember that the underlying causes of this rise are the same in all the affected countries, though in each there may be very important modifying factors, such as the local results of wars, immigration and emigration. Thus the high percentage of old people in Eire is at least in part due to the constant emigration of her younger individuals, while on the other hand in a receiving country like New Zealand, it has been estimated that the proportion of 9·1 per cent. which obtained in 1952 may have actually decreased to 8·4 per cent. by 1972, on the basis of an annual immigration intake of 5,000[3]. Factors such as these are probably the cause of very interesting differences in the rate of ageing of countries with a similar cultural background, as is shewn in the following table from Professor Sauvy[4]:—

TABLE I

| Percentage of Sexagarians | Year reached in: France | Sweden | England and Wales | Germany | Italy |
|---|---|---|---|---|---|
| 8 .. .. | 1790 | 1850 | 1910 | 1911 | 1860 |
| 18 .. .. | 1964 | 1955 | 1962 | 1964 | 1988 |

These countries do not have a random distribution over the globe, but are those that have adopted "Western" or scientific ways of life, particularly in matters of medicine and public health. The increase in the numbers and proportion of old people in their populations is due to the simultaneous operation of two independent, but synergistic, processes. The one, which is well recognised, is our recent control over such natural processes of denudation as the infectious diseases of early life, which increases the expectation of life at birth and so allows more individuals to live long enough to reach the old age period. The other factor, whose importance is not as a rule nearly so well recognized, is the decline in birth rates which has been more or less continuous since the second half of the last century. The smaller intake into the population at birth entails, for a considerable period of time, an increase in the proportion of those of greater age, so that in place of the normal smooth population pyramid there may now be less children aged 0 to 5 years than aged 5 to 10 years, a reverse of the usual state of affairs. This is the beginning of the well-known "bulge" in the population pyramid, and since it takes an individual 65 years to reach the old-age period, the maximum effect will be correspondingly delayed and will continue until the bulge has worked itself out at the top. This introduces a factor of enormous social importance. Its degree may be gauged from the fact that in Great Britain in 1871, 295 babies were born to every thousand married women, while by 1951 the figure had fallen to 108. It is this factor which is mainly responsible for the increase in the proportion of old people as distinct from their total numbers, the obverse effect of which is a decrease in the numbers of the younger generation available to look after the old people. Thus Titmuss[5] has suggested that as many as 20 per cent. of the old people of Great Britain at the present time may be without children available to care for them.

The truth is that this demographic change is the inevitable result of letting loose on a community the forces of modern scientific medicine, and in the course of time it is bound to affect vast areas of population, as in Asia, where at the moment the process is only beginning. If it is important to realize that we are witnessing a process that is likely in the course of time to affect the whole world, it is equally important to realize that it is only of very recent origin. This is well illustrated by the history of Great Britain, where in spite of a steady growth in total population during the Victoria era, the proportion of old people remained steady at 5 per cent. until as late as the 1921 census, when it rose for the first time to 6 per cent. reaching 7 per cent. in 1931 and almost 11 per cent. by 1951, and is now probably somewhere around 13 per cent. The tide is therefore still on the make and is likely to reach its height in another twenty years or so, when the five million old people of 1951 may have risen to eight or nine million, or perhaps 18 per cent. of the projected population.

The speed of development is well illustrated by the history of the International Association of Gerontology, which was founded as recently as 1950 at Liège in Belgium under the Presidency of Professor Brull. Membership is open to countries that possess societies devoted to the study of old age, and there were twelve foundation countries—Belgium, Denmark, Eire, Finland, France, Great Britain, Italy, the Netherlands, Spain, Sweden, Switzerland, and the United States of America. The original membership has now expanded to twenty-four countries. Every country in Western Europe is now a member and within the Iron Curtain Hungary and Roumania have joined. Of the greatest general significance is the fact that four countries in Latin America are now represented—Argentine, Mexico, Peru, and Venezuela, and indeed a special Pan-American conference was held in Mexico City in 1956, which is a clear indication that this part of the world is beginning to be affected by this population change. Of at least equal significance for the future is the fact that Japan—the first of the countries of Asia to adopt a Western way of life in medicine and public health—is now a member with two flourishing societies in Tokyo and in Osaka. It is clear, therefore, that the process of "growing old" is rapidly becoming one of world-wide interest.

### HEALTH OF THE OLD

We may now consider briefly one further general question—it is often said that since there are many more old people alive today than of yore, their health might be expected to be better than it used to be. In the absence of accurate information as to the real level of health of our present old people, let alone of those of a century ago, it is quite impossible to give a definite answer, but there are pointers which give disquieting results. Here I would refer to figures provided by the Government Actuary to the Phillips Report to the Chancellor of the Exchequer on the financial problems of ageing[6]. Three lines are abstracted, giving the percentages of the old age population in the different quinquennia of old age at censuses since 1851.

TABLE 2

| | 65 | 70 | 75 | 80 | 85 |
|---|---|---|---|---|---|
| 1851 .. .. | 39 | 30 | 18 | 9 | 4 |
| 1931 .. .. | 43 | 29 | 17 | 8 | 3 |
| 1951 .. .. | 38 | 30 | 19 | 9 | 4 |

If one compares the figures for 1931 and 1951 it will be seen that in the latter year there was a fall of 5 per cent. in the proportion of those in the first quinquennium 65–70, the balance being distributed among those reaching greater ages. There was therefore a considerable improvement in mortality rates in the twenty years. This period, however, was marked by the introduction of the sulphonamides and the antibiotics which for the first time in human history made it possible to control one of the major killing agents in old age—i.e. acute respiratory infections, such as pneumonia. Society in this period has undoubtedly paid a price for the gain in individual expectation of life, for the nursing of an old person over the short period of time occupied by an acute respiratory infection causes no great domestic stress. On the other hand, the nursing of an old person with a degenerative condition such as a stroke or a traumatic condition such as fractured femur can impose the greatest strains, due to the length of time for which they may have to be carried. There is little doubt that as a result of our success in controlling the acute infectious diseases of age, what may be termed the "duration of final incapacity" of the old age population as a whole has increased. We might nevertheless be inclined to view this state of affairs with complacency until we make a comparison between 1951 and the state of affairs in 1851, which are virtually identical. This is indeed disturbing. Anyone who was eighty years old in 1851 had been born in 1771, and anyone born in 1771 who subsequently reached the age of 80, was extremely unlikely to have done so by virtue of any special help he may have received from my profession. He then had to run the gamut of the diarrhoeas of infancy, the acute infections of childhood and adolescence, and in later life could obtain no help from abdominal surgery, to mention only a few factors. The old age structure of 1851 represents the survival of the fittest in the biological conditions of a modern community, and it is somewhat disturbing that the greatest triumphs of modern times, the sulphonamides and the antibiotics, have not only failed to improve on this figure, but have merely buttressed up to 1851 standards a previously lowered state of health. This certainly gives us food for thought. My own personal view is that we have in our old age population more than one biological race—that we still have and in fact can easily recognize those remarkable individuals who make old bones without any illness until the final one, but that we also have those who are only in the old age group because they have been saved from extinction at an earlier period, and a comparison of the figures of 1851 and 1931 makes one wonder whether the health of our present old age population—taken as a whole—is in fact equal to what it was in 1851.

### WHEN DOES OLD AGE BEGIN?

This, then, is the general framework of events within which we must discuss the individual processes and problems of ageing. I have dealt with it at some length not only for its intrinsic interest, but because it helps one to understand that many of the problems are as much social as individual. In approaching the individual problems of ageing there are two general factors which seem to be to be of general importance. The first concerns the time of onset of old age, and the second its sexual incidence. The age of onset of old age in this country is officially taken to be 60 for women and 65 for men, those being the ages at which contributory pensions become payable. It is essential to stress the fact that these arbitrary figures, however convenient they may be from an administrative point of view, have no biological validity. With the steady growth during this century of the tradition of retirement at a fixed age, there has come to be a feeling that these birthdays represent some deep fundamental change in the rhythm of life, which most definitely is not the case, and there are three aspects of the onset of ageing which are worth stressing. In the first place it is quite impossible to lay down any period of time applicable to all individuals, for there is an enormous range of individual variation. In the second place it is impossible to lay down a period of time applicable to all activities, for the old-age onset for the ski-jumper, for example, is very different from that for the bird-watcher. In the third place, there is variation in the decline of particular functions. Hearing for very high-pitched sounds is most acute at the age of ten, after which the process of decline begins. After making all allowances for individual variation, the average individual can probably look forward to a further five or even ten years beyond the official ages, during which he or she can make an active contribution to the total effort. It is of some interest to compare the official ages of retirement with those followed by individuals who are self-employed at an occupation which gives them satisfaction. Thus in the case of housekeeping for women, 50 per cent, of women at the age of 60 are engaged in the entire care of their household, and the figure is unchanged at the age of 70. By 75 it has fallen to only 40 per cent. but thereafter the effects of age become increasingly apparent so that at 80 only 18 per cent. and at 85 only 4 per cent. are so occupied[7]. These figures illustrate the discrepancy between the official and the natural onset of old age, for it would appear for most individuals real old age does not come to dominate their lives till after the age of 70, and even then there is enormous individual variation. Whatever may be the drawbacks of housekeeping, the old woman looking after a household has the satisfaction of knowing that her existence and her work is necessary to others—a matter of the greatest importance to old people. It is for this reason that so many old people try to carry on longer than perhaps they should, as may be seen from the very odd fact that the proportion of old women partially engaged in housekeeping is at its highest in the period 80 to 85, when slightly more than half of the women do something to help in the house. This ability to feel continuously useful, to have something at hand to do, may perhaps be one of the unknown factors responsible for the undoubted capacity of the woman to outlive the man. At any rate, the figures do help to demonstrate something that it is essential to remember, that in old age it is good to have something to live for and that it is not a period that is made either happier or healthier by the cessation of activity. Indeed, a long acquaintance with old people living at home has made one realize the extent to which those who make a success of old age do continue to demand from their body all that it can give, which I regard as one of the prescriptions for successful old age.

Thus I was very impressed when I was crossing the Atlantic in the Spring of 1955 to meet an old man of eighty. When I first met him, he was hobbling about on deck with two sticks and with some difficulty, but he was on deck every day of the passage except for one when we ran into a real gale. This old man was a widower who was going out to Boston to visit his married daughter. He had osteo-arthritis of the hip-joints, and when I asked him how he managed he replied that it had been a big blow when his wife died as she used to help him to dress, in which his stiff hips caused him great difficulty. However, he said that he had got over it by having a wall fixture made to which he could hold while putting on his trousers, for which purpose he had also had a special pair of tongs made.

It is this refusal to strike the flag in the face of physical infirmity that is essential to successful old age.

### SEX DIFFERENCES IN OLD AGE

One other general fact about human ageing may now be mentioned, and that is the difference between the sexes. It is well-known that women tend to outlive men, though its extent is perhaps not always recognized. Where as in the sixties there are roughly equal numbers of men and women, there are no less than three times as many women as men in the nineties, and in centenarians the proportion may be as much as five to one. Seeing that their mortality rates are so much better, one would expect that old women would have corerespondingly better health than old men, but all investigations have shown that the reverse is the case. Women in old age have a consistently higher morbidity rate than do men for all ailments that have no inherent sexual bias. The reason for this is quite unknown, and it does not appear to be related to the chromosomal differences, for in birds where the male has the even chromosome distribution, the female still appears to live longer. Although the biological interest of this feature is great, its social importance is at least equal. It is the cause of the great shortage of hospital accommodation for old women, for not only do they have more illnesses, but by living longer they require more nursing, and the maintenance of the health of old women becomes therefore a problem of great practical importance.

### THE CARE OF OLD PEOPLE IN THEIR OWN HOMES

Hitherto we have been dealing with old age from the point of view of whole populations, and it is now time to turn our attention to the standpoint of the affected individual. I will base what I have to say on a recent statement of Government policy in this country, in which the Ministry of Health has indicated its complete agreement with the conclusions of the Guillebaud Committee that "The first aim should be to make adequate provision wherever possible for the treatment and care of old people in their own homes". This is an extremely interesting statement, and in adopting this policy Great Britain is indeed a pioneer. As soon as a country becomes conscious for the first time of its old-age problem, the first reaction is always away from the individual's home, towards the provision of hospital treatment, and the erection of special dwellings such as bungalows, flats and hostels. The sheer scale of the problem is never realized at the outset, and not until later does it become clear that these services—whether voluntary or official—can cater only for the minority of old people. There can be no doubt whatever of the wisdom of the British policy, but if it is to be successful, the problem of institutional care will need to be re-examined. The reason is that a considerable proportion of old people are now without relatives in the younger generation available to care for them. An official statement admits that at the present moment "two-thirds of the Hospital beds in this country occupied by those over sixty-five are taken by the single, widowed and divorced, and the majority of the patients of this age-group in the mental hospitals are single people"[8]. These people form a group that I have labelled the "isolates" who are important since they prevent a proper use being made of the Hospital beds available for old people, owing to the impossibility of discharging them when they are otherwise fit to leave hospital. We must therefore inquire into the factors conducive to the maintenance of the health of old people at home.

1. I would say that the first requirement is to take all necessary national steps to keep our old people as fit as possible, so that they may be enabled to maintain their independence for as long as possible. To this end the first requirement on a national scale is undoubtedly to see that they have adequate spectacles. One of the most important physiological changes in age is that information derived from the eyes becomes steadily more important than it was in youth. Such things as balance can no longer be maintained in the dark with the ease of youth, and all investigations have shown the increasingly important part played in old age by visual control of muscular activities. This is at a time in life when the eyes themselves undergo structural defects, and the provision of adequate spectacles becomes therefore a matter of necessity. Prior to the introduction of the National Health Service, the situation was deplorable, for as many as one-third of the old-age population admitted that their spectacles were useless, and it was a safe guess that at least one-half would have derived benefit from proper spectacles. The immediate run on spectacles following the inception of the Health Service illustrates the extent of the demand.

2. The other matter that deserves interest on a national scale is attention to the feet of old people. I never speak on the subject of old age without referring to this subject, which is in my opinion a challenge to public health. Painful and deformed feet occur frequently in both sexes, but are more frequent in women, where they are the cause of much domestic misery and incapacity, all the worse in that such an isolated complaint may grossly impair the vigour and activity of an otherwise healthy individual. The extent of the complaint is almost unbelievable. In a recent survey of old people in Sheffield[9], painful feet ranked fourth in the list of causes of restricted mobility; in Wolverhampton 26 per cent. of the men and 45 per cent. of the women had trouble with their feet[7], while in a special survey at Rutherglen in Scotland, of 119 elderly people, only eight were considered to have normal feet. There can be no doubt therefore as to the necessity for attention to this matter, and indeed the provision of adequate chiropody would be of immense assistance to our old folk. Prevention is of even greater importance, and the wearing of tight socks and shoes in younger years seems to be a very relevant factor. It was quite surprising in a recent visit to Australia to note how the incidence of painful or deformed feet seemed to decrease as one went northward into warmer parts, where it was the custom for the young to go barefoot or wearing only the lightest footwear in contrast to the colder parts with a climate like our own where for at any rate part of the year stout leather footwear was a necessity.

3. In addition to good vision and sound feet there is little doubt that good hearing means a lot to old people, a fact of all the more importance in that a decline in auditory activity seems to be part of the normal ageing process. The provision of hearing aids under the National Health Service has undoubtedly been a great boon. Apart from these three features which are really matters of public health, I think that the most useful general advice one can give old people is to use body and mind to the full. It has to be remembered that with the best will in the world it is impossible to transform the physical state of a man of seventy into that of a youth of twenty, and that various weaknesses and disabilities are the inevitable accompaniment of ageing. What matters is the continuance of the will to get the most out of the body, and I cannot help saying how enormously I admire the vigour and tenacity of our old people in this respect. I have already mentioned one instance of the over-riding of a physical handicap. The Sheffield survey[9] gives us a statistical indication of how frequently this occurs and of its social importance.

TABLE 3.

**Mobility and Assessment of Fitness**

| Survey | Unrestricted Mobility (per cent.) | Self-Assessment as fit (per cent.) | Physician's Assess- as fit (per cent.) |
|---|---|---|---|
| Sheffield (men.) .. | 71·2 | 61·4 | 26·2 |
| Wolverhampton (men) .. .. | 70·0 | — | 35·4 |
| Sheffield (women) .. .. | 54·9 | 48·7 | 23·0 |
| Wolverhampton (women) .. .. | 63·5 | — | 19·4 |

It will be seen that the subjects tended to rate their own state of health better than did the examining physician, and in fact the number of old people who regarded themselves as fit was more than twice as large as those considered to be fit by the physician. This is all to the good.

The other feature on which all are agreed is that there is no other period in life in which physical health is so dependent on mental health as in old age. The two great enemies are boredom and loneliness. It is here that the woman is usually so much better off than the man, for not only is she so often free from the sudden contraction of interest that may follow compulsory retirement, but in addition as a grandmother she has easier access to a combination of occupations and human interest than the male. One of the prescriptions for ideal old age is undoubtedly a consuming hobby. It is within this framework that the whole problem of the continued employment of our older men and women has to be examined.

### ACCIDENTS AT HOME

We may now ask "what are the major occupational risks of living at home?" The liability to injury from falling is without doubt the major risk, and it constitutes a problem of the greatest social importance, as well as clinical interest. Ninety per cent. of fatal falls occur after the age of 65, and 65 per cent. of fatal domestic accidents fall within the same period. Women are more frequently affected than men, and by the age of 80, whereas 80 per cent. of women have had one or more falls, only 40 per cent. of men have been so affected. In 1956 there were 7,000 fatal domestic accidents in this country. Of these 5,000 occurred after the age of 65, and 4,000 after the age of 75, the great majority of the domestic accidents being falls. The reasons are manifold, as are the effects. A fall in old age may be immediately fatal; it may lead to fracture, particularly of the neck of the femur with much and persistent disability; it may initiate a general senile breakdown, and finally the fear of one of these results may greatly impair an old person's comfort and sense of security. The reasons are obscure, but falls can be classified into certain types. Some are due to an attack of vertigo, some are due to a liability in old age to trip up over trivial domestic excrescences, such as a rug; some are due to the adoption of dangerous positions, some are due to the dark, and some seem to occur without reason; but all falls in old age are conditioned by one further factor. Old people will commonly assert that "once you are going, you've got to go"—meaning that once a fall has started the old person cannot make those rapid adaptive movements available to the younger individual. The reasons for this are obscure, but there is undoubtedly a slowing up of the cerebral factors that control the maintenance of posture, so that by the time a decision has been made as to the appropriate movement necessary to remain upright, the act of falling has progressed so much further that the decision is already antiquated. In ageing there is a loss of nerve cells from the brain, which seems particularly to affect the Purkinje cells of the cerebellum[10], and since this part of the brain is intimately concerned with the control of muscular movement, this may well be an important factor. As I have already indicated, the subject is one of the greatest clinical interest, but it is also of equal practical importance. In order to minimize the risk of falls and accidents in the house a few common-sense precautions are advisable. There should be no slippery surfaces and in particular no rugs or mats lying loose on a smooth oilcloth. There should be adequate illumination, particularly in places which are usually deficient, such as landings and bends in staircases, and the kitchen sink, and the use of nightlights in the bedroom is often useful. There should be adequate supports or handrails on the stairs, and in the bathroom and lavatory. Certain movements should be discouraged or abolished. The practice of climbing on domestic furniture, which is a favourite activity of some old women in order to reach curtain rings and so on, should be forbidden; and so also should be the practice of working at an object above the head with the head thrown back, such as the top shelf of the larder. It is remarkable how many domestic accidents in old women are due to this cause alone. Finally there should be no reluctance to make use of sticks. Allied to the danger of falling is a further occupational hazard of those old people living at home which is rarely thought of—fear of traffic. This is a purely senile manifestation and does not occur until about the age of 75, but thereafter it becomes an increasingly frequent cause of restricted mobility. One result is the inability to make use of public bus services and the other is the fear—usually well founded—of crossing the road when the traffic is busy.

### LONELINESS

The mental hazards of home life for old people are mainly those that follow loneliness or boredom. Loneliness is a terrible handicap for many old people and the evidence seems to be clear that although the origin of the emotion is personal in the sense that confronted with the same situation one will feel lonely and the other not, yet in the course of time it does lead to a depression of mental and also of physical vigour. It is all the more liable to occur in modern times with the decline in the number of children and the separation of the generations that results so often from the development of the modern housing estates. So important is loneliness that, as is well-known, it is nowadays put in the forefront of the domestic services that should be provided for old people.

It is impossible to consider the policy of maintaining as many as possible of the old people at home without dealing with the social implications. Old folk at home may be broadly divided into the two classes of those with and those without, family contacts. To the latter group belong the isolates, whom I have already mentioned—those who have no-one at hand to help in time of trouble, and who lead completely isolated lives. This group is of the utmost practical importance, for in illness their very isolation gives them a precedence for the available services over those with family connections, which is often very unfair. Whilst it is true that they must be given all the domiciliary help needed, as by home helps, district nurses, "meals on wheels" and so on, help for the old must not stop there, and regard must be paid to the assistance of those who at the moment are carrying much the biggest share of the load—the family. Unfortunately, it is only of recent years that the importance of the family in the welfare of the aged has come to occupy its rightful place, and many of the social improvements of recent years having been planned without regard to the family—have unintentionally made their position more difficult. In considering the relation of the family to their old people there are two essential points to remember:

1. That some of the families have their lives overburdened by the care of one or more aged relatives, and that as recently as 1947 no less than 7 per cent. of the old people were causing a severe strain on the domestic life of their younger relatives.

2. The method that is universally regarded as ideal, at any rate in Great Britain, both by the old people and by the younger generation, is for the two generations to live close but independently. It is not without significance that of widowers and widows living alone, no less than half have children living close and all investigations whether in the Midlands, Bethnal Green[11], or elsewhere, have agreed on the importance in the care of the aged of the family unit which is spread over more than one house. It is here that such recent social developments as the big housing estates and the increasing employment of married women have made the work of the family more difficult. Yet when one remembers that certainly ninety per cent. and probably as many as ninety-five per cent. of old people are living in their own homes, it will be clear that any failure by the family to continue to carry its previous share of the burden would present the community with an impossible task. That is where we can derive comfort from the declared policy of the Ministry of Health to maintain old people at home for this will inevitably result in help having to be given to the hard-pressed family.

### BIBLIOGRAPHY

[1] Miss Margery Fry, (1955): "*Old Age in the Modern World*", p.4. E. S. Livingstone, Edinburgh and London.

[2] Durand, J. D. (1955): "*Old Age in the Modern World*", p.33. E. S. Livingstone, Edinburgh and London.

[3] "*Some facts concerning old people in New Zealand.* Department of Health, Wellington, N.Z. (1955).

[4] Sauvy, A. (1955): "*Old Age in the Modern World*". P. S. Livingstone, Edinburgh and London.

[5] Titmuss, R. M. (1955): "*Old Age in the Modern World*". E. S. Livingstone, Edinburgh and London.

[6] Report on the Economic and Financial Problems of the Provision for Old Age. Cmd. 9333. London. H.M.S.O. (1954).

[7] Sheldon, J. H. (1948): "*The Social Medicine of Old Age*". Oxford Press, Oxford and London.

[8] Boucher, C. A. (1957): *Minutes of Health Reports on Public Health and Medical Subjects.* No. 98. p.4.

[9] "*The Health of the Elderly at Home*". (1955). W. Hobson and J. Pemberton. London. Butterworth.

[10] Bourne, G. (1956): "*Modern Trends in Geriatrics*". Butterworths. London. p.31.

[11] Tounsend, P. (1957): "*The Family Life of Old People*". Routledge and Kegan Paul, London.

